# A Machine Learning Based Dose Prediction of Lutein Supplements for Individuals With Eye Fatigue

**DOI:** 10.3389/fnut.2020.577923

**Published:** 2020-11-13

**Authors:** Juntao Kan, Ao Li, Hong Zou, Liang Chen, Jun Du

**Affiliations:** ^1^Nutrilite Health Institute, Shanghai, China; ^2^Department of Bioinformatics, WuXi NextCODE Genomics, Shanghai, China

**Keywords:** dose prediction, machine learning, XGBoost, lutein supplements, eye fatigue

## Abstract

**Purpose:** Nutritional intervention was always implemented based on “one-size-fits-all” recommendation instead of personalized strategy. We aimed to develop a machine learning based model to predict the optimal dose of a botanical combination of lutein ester, zeaxanthin, extracts of black currant, chrysanthemum, and goji berry for individuals with eye fatigue.

**Methods:** 504 features, including demographic, anthropometrics, eye-related indexes, blood biomarkers, and dietary habits, were collected at baseline from 303 subjects in a randomized controlled trial. An aggregated score of visual health (VHS) was developed from total score of eye fatigue symptoms, visuognosis persistence, macular pigment optical density, and Schirmer test to represent an overall eye fatigue level. VHS at 45 days after intervention was predicted by XGBoost algorithm using all features at baseline to show the eye fatigue improvement. Optimal dose of the combination was chosen based on the predicted VHS.

**Results:** After feature selection and parameter optimization, a model was trained and optimized with a Pearson's correlation coefficient of 0.649, 0.638, and 0.685 in training, test and validation set, respectively. After removing the features collected by invasive blood test and costly optical coherence tomography, the model remained good performance. Among 58 subjects in test and validation sets, 39 should take the highest dose as the optimal option, 17 might take a lower dose, while 2 could not benefit from the combination.

**Conclusion:** We applied XGBoost algorithm to develop a model which could predict optimized dose of the combination to provide personalized nutrition solution for individuals with eye fatigue.

## Introduction

Eye fatigue also known as asthenopia, is a common condition in both adults and children, which can be caused by various reasons, especially the intensive use of electronic products e.g., computers, cell phones and iPads (1). Nutritional intervention that provides certain benefit to ocular health has been researched for decades (2). Lutein and zeaxanthin are known to protect retina because of their anti-oxidant nature and ability to absorb high-energy blue light produced by visual display units (3). Moreover, the two ingredients, when combined with other botanical ingredients with rich anthocyanin, were reported to relieve eye fatigue (4). So far, however, most strategies for preventing or reducing the incidence of the symptoms are based on “one size fits all” public health recommendations to the whole population.

Based on anthropometrics, blood biomarkers, dietary habits and physical activities, the solution of personalized nutrition has been tailored to meet specific nutritional needs these years (5). Machine learning as a field of computer science adopts computer algorithms to identify patterns in large datasets with numerous variables, which can be used to predict data-based outcomes (6). Machine learning algorithms, including random forest (RF), extremely randomized trees (ET), extreme gradient boosting (XGBoost), and gradient boosting decision tree (GBDT), usually establish a model from test inputs to make predictions or decisions based on the data (7). Nowadays, machine learning techniques have proven to be highly effective for prediction of response to methotrexate and antidepressant medication, and diagnoses of pediatric diseases and upper gastrointestinal cancer (7–10).

A novel combination of lutein ester, zeaxanthin, extracts of black currant, chrysanthemum and goji berry was previously developed, showing protective effects on eye fatigue, dry eye, and macular function in a randomized controlled study (RCT) (11). Here using machine learning technology, we tried to, by leveraging several algorithms, predict the optimal dose of the combination based on the features collected in the RCT, in order to provide personalized nutrition solution for the future in real world.

## Methods

### Clinical Trial

The RCT was conducted in Aier Eye Hospital, Shanghai, China. A total of 360 subjects with eye fatigue were initially enrolled in the study at baseline, and randomized into 4 arms (arm 1: placebo; arm 2, test product containing 6 mg of lutein; arm 3, test product containing 10 mg of lutein; arm 4, test product containing 14 mg of lutein) to receive either test products or placebo orally once daily for 90 days. Each subject had a total of 3 visits at baseline (visit 1, V1), 45 days (visit 2, V2), and 90 days (visit 3, V3) throughout the study. During the study, 42 subjects withdrew after the first visit and 15 more subjects withdrew from the study after the second visit, leaving 303 subjects in the statistical analysis. Scores of eye fatigue symptoms (EFS), visuognosis persistence (VP), macular pigment optical density (MPOD), and Schirmer test (ST) were collected at all 3 visits, while anthropometrics, physical activities, food frequency questionnaire (FFQ), optical coherence tomography (OCT), and blood biomarkers, including blood lipids, liver and renal function, were collected at V1 and V3. Informed consent was obtained from all subjects. This study was approved by the Institutional Review Board (IRB) of Shanghai Nutrition Society and registered at chictr.org.cn (ChiCTR1800018987). More details on the study design were published previously (11).

### Machine Learning

**Figure 1:**
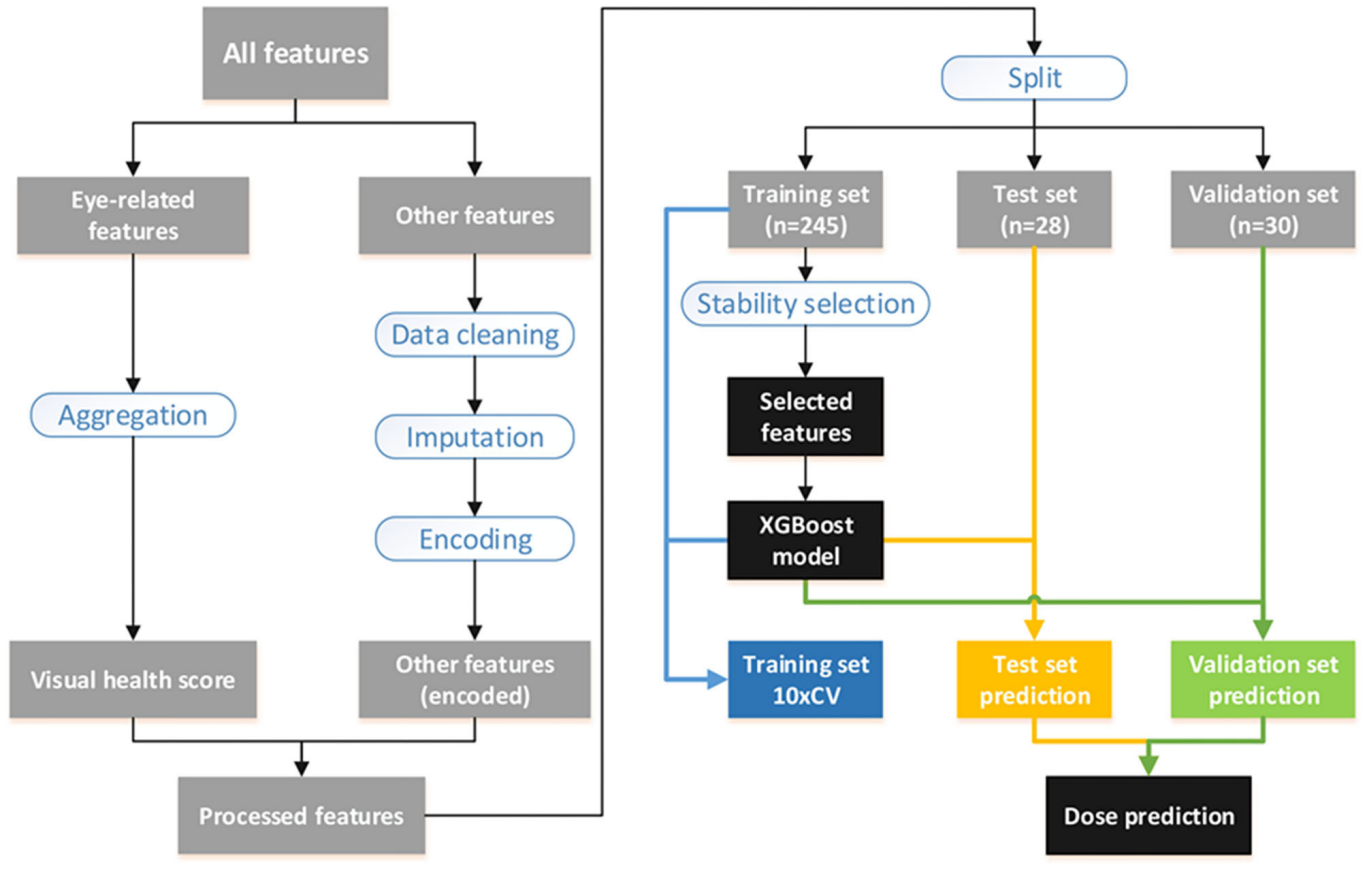
Workflow overview.

A total of 504 features collected from 303 subjects were used in the model building. The abbreviation of finally selected features was listed in Table 1. A full list of the 504 features were shown in Supplementary Material 1. Four eye-related features, including total score of EFS (TSEFS), VP, MPOD and ST, were aggregated to a score named visual health score (VHS). Other features were preprocessed with cleaning and imputation before encoding. All 303 subjects were split into 3 subsets, i.e., the training, test and validation sets. Training features were selected by stability-selection method, and a final model was built by XGBoost using selected features. Model performance was evaluated in the test and validation set, and optimal dose for each subject was predicted with the validated model. The workflow of model building was shown in Figure 1. Machine learning techniques were implemented in Python 3 (Python 3.7.3) using the package scikit-learn (0.21.3), xgboost (0.90), xlrd (1.2.0), and numpy (1.17.0).

**Table 1:** Abbreviation list.

**Abbreviation**	**Full Name**	**Abbreviation**	**Full Name**
BFR	Body fat rate	RDWCV	Red cell distribution width-coefficient of variation
CRE	Creatinine	RDWSD	Red cell distribution width-standard deviation
Dairy3	Whole milk	RTLC	Retinal thickness of left eye-central average
DBP	Diastolic blood pressure	RTLCMn	Retinal thickness of left eye-central minimum
FFM	Fat free mass	RTLU6	Retinal thickness of left eye-up 6 mm to center
Fruit3	Banana	RTRD3	Retinal thickness of right eye-down 3 mm to center
Fruit8	Pear	SBP	Systolic blood pressure
HDL	High density lipoprotein cholesterol	ST	Schirmer test
LDL	Low density lipoprotein cholesterol	TG	Triglyceride
LYMPHC	Lymphocyte count	TSEFS	Total score of eye fatigue symptoms
LYMPHP	Lymphocyte proportion	Veget4	Cruciferous vegetables
MCHC	Mean corpuscular hemoglobin concentration	Veget7	Other vegetables
MCV	Mean corpuscular volume	VF	Visceral fat
MPOD	Macular pigment optical density	VHS	Visual health score
PDW	Platelet distribution width	VP	Visuognosis persistence
PLT	Blood platelet count	WBC	White blood cell count

### Data Preprocessing

Data with any obvious writing mistake or missing value were replaced and imputed by mean of all non-missing cases of the corresponding feature. Then data were encoded according to different types: (1) continuous data remained the same; (2) discrete data were encoded by one-hot method; (3) questionnaire data with multiple choices were multi-hot encoded. All 303 subjects were split into 3 subsets of training (*n* = 245), test (*n* = 28), and validation (*n* = 30) by an approximate ratio of 8:1:1. Subjects were stratified by dose in test set while randomized in validation set. Both sets were set aside during feature selection and parameter optimization, and they were used for model evaluation only.

### Feature Selection and Model Evaluation

XGBoost, AdaBoost, and ElasticNet algorithms were applied in the model building. All baseline features were used for training, while VHS of post-intervention was used as the target for prediction. Pearson's correlation coefficient (PCC) between real and predicted VHS was used to evaluate model performance. Features were selected using stability-selection method (12). In brief, the method took different subset of samples to train model with different complexity (lambda parameter) before outputting a set of stability scores for each feature. Tree depth from 2 to 10 of XGBoost was used as lambda parameter for stability-selection, and the threshold was determined by: (1) keeping reasonably less features; (2) not lowering average PCC of 10× cross validation (10×CV) in training set. Finally, the model was retrained using selected features with the optimized parameters before it was used to predict VHS of post-intervention in test and validation sets where PCC was calculated for model evaluation.

### Dose Prediction

Twenty eight subjects in test set and 30 in validation set were used for dose prediction. It was supposed that each subject took 3 different doses (containing 6, 10, 14 mg of lutein). The validated model was used to predict VHS at V2 of each subject that underwent three separate doses. The dose with the highest VHS was recognized as the optimal option.

## Results

### The Botanical Formula Improved Eye Fatigue

**Figure 2:**
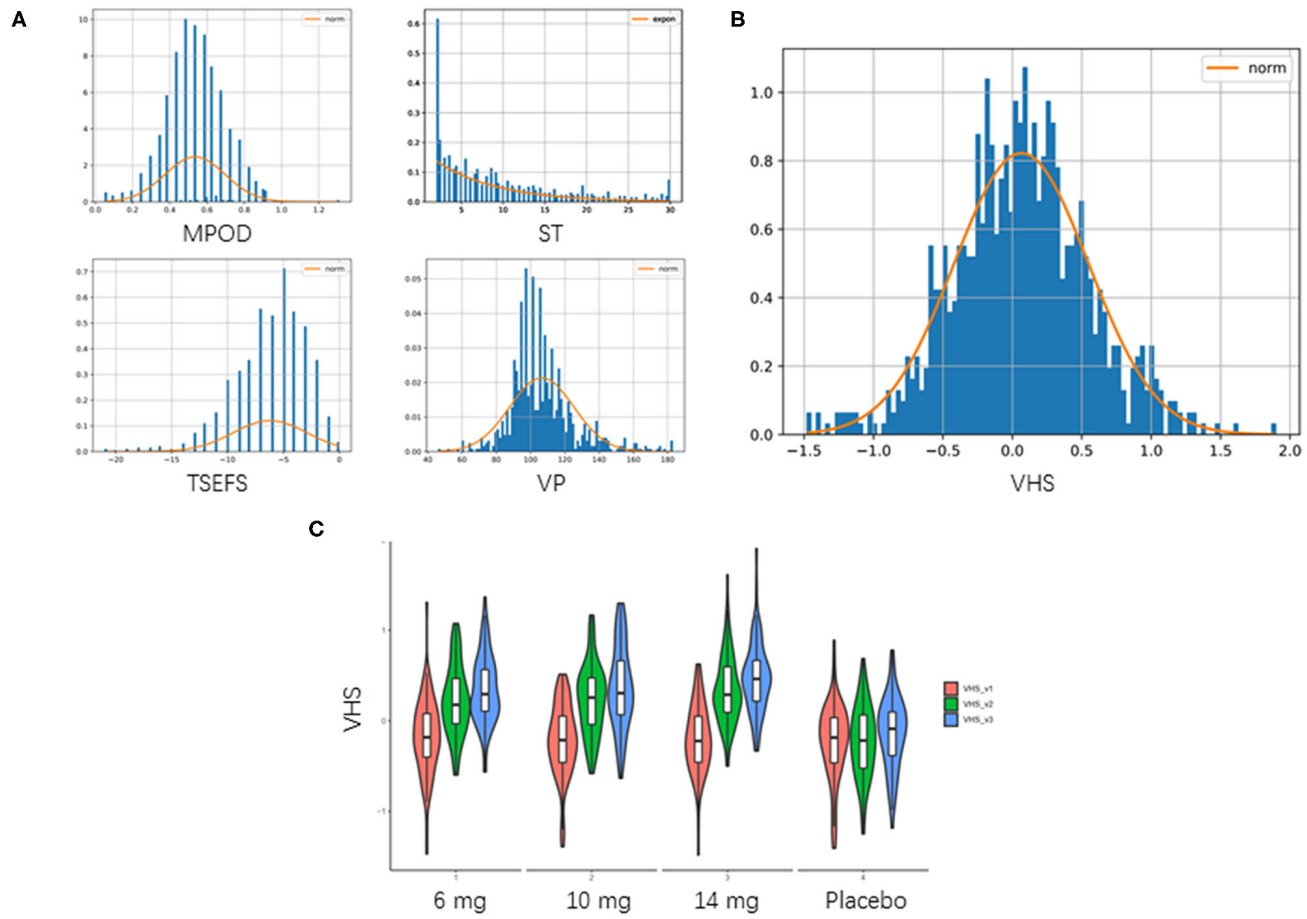
Aggregation of visual health score. **(A)** Distribution of 4 eye-related features. TSEFS, VP and MPOD were Z-score transformed as they followed normal distribution approximately. ST was not normally distributed, then it was min-max scaling transformed. **(B)** Distribution of VHS. VHS was taken as mean of these 4 transformed features, which followed a normal distribution of N (0.064,0.485) with *P*-value of Kolmogorov–Smirnov test = 0.557. **(C)** Violin plot of aggregated VHS. Red: VHS at V1 (0 day); green: VHS at V2 (45 days); blue: VHS at V3 (90 days).

Baseline characteristics, including demographic, anthropometrics, and eye-related indexes, were shown in Table 2. An aggregated score, VHS, was developed from 4 eye-related features, including TSEFS, VP, MPOD and ST, to represent an overall eye fatigue level, following a normal distribution (Figures 2A,B). Formula intervention with different doses significantly improved VHS at V2 and V3 compared to placebo arm (Figure 2C). Since there was a slight, but not significant, increase of VHS at V3 compared to that at V1 in placebo arm, while the values of VHS at V2 and V1 were comparable, the VHS at V2 was chosen as a quantitative measurement of eye fatigue improvement.

**Table 2:** Baseline characteristics.

**Characteristics**	**Data *N* = 303**
Ethnicity (Han Chinese)	303 (100%)
Gender (male)	67 (22.1%)
Age (year)	38.2 ± 8.3
Weight (kg)	66.1 ± 14.2
Height (cm)	164.9 ± 8.1
Body fat rate (%)	47.5 ± 11.1
Total score of eye fatigue symptoms	7.6 ± 3.6
Visuognosis persistence (seconds)	99.5 ± 15.7
Macular pigment optical density	0.50 ± 0.17
Schirmer test (mm)	7.6 ± 6.9

*Data were presented as mean ± standard deviation or frequency (%)*.

### XGBoost Successfully Selected Key Features and Predicted Eye Fatigue Improvement

**Figure 3:**
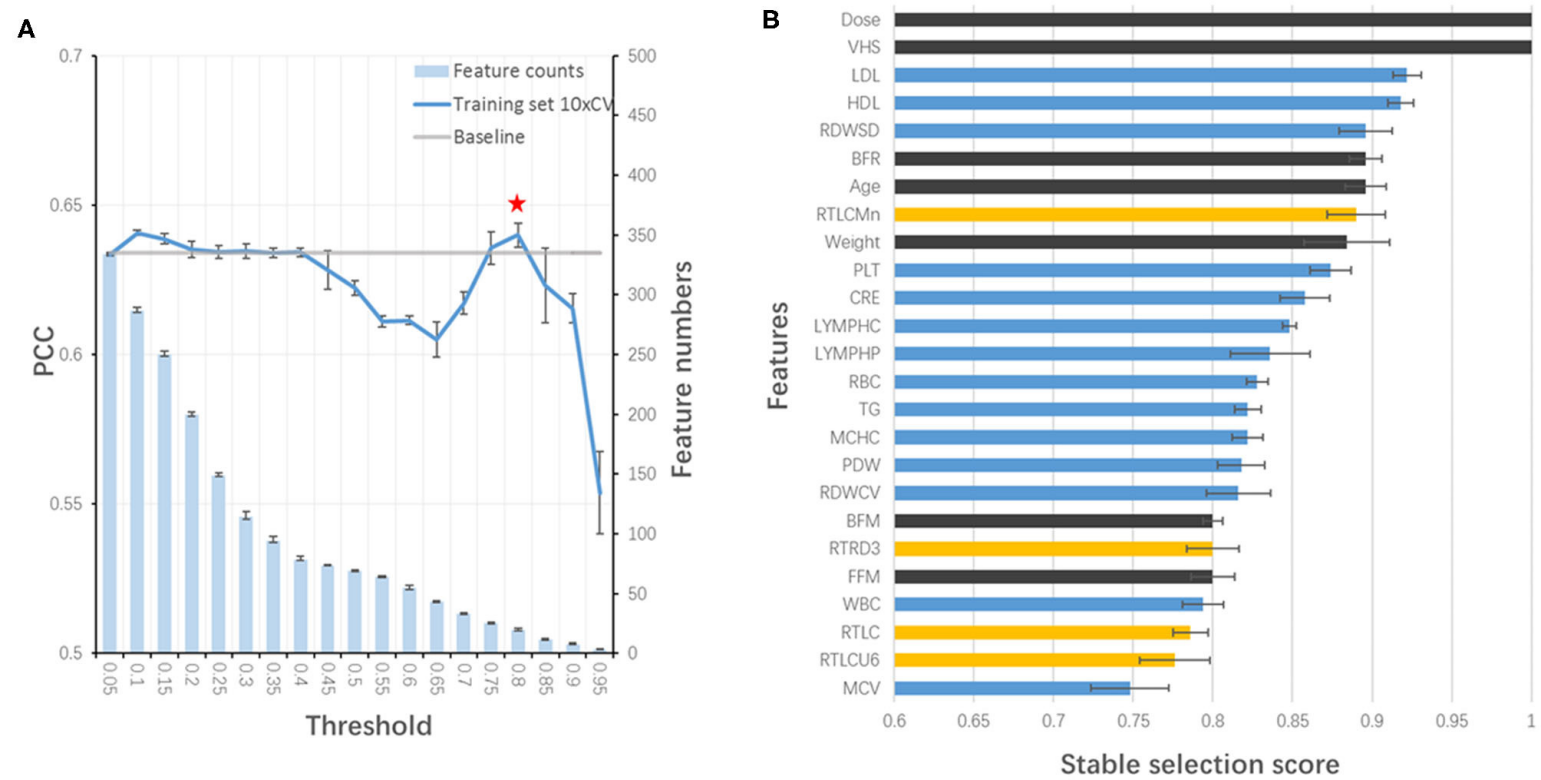
Stability-selection of features. **(A)** Stability-selection with different threshold. Star denoted chosen threshold (0.8). Gray line: baseline PCC without stability-selection; blue line: 10xCV PCC in training set using different selected features; blue bar: feature numbers. **(B)** Selected features. Blue, blood test features; yellow, OCT features; black, other features.

All features at baseline were used as training features to construct a baseline regression model to predict VHS at V2 by applying different algorithms. Among them, XGBoost (PCC of training set =0.618) outperformed others such as AdaBoost (0.604) and ElasticNet (0.412), and therefore, was selected for the model building. Next, stability-selection was applied to select key features with getting better performance. A total number of 25 key features (out of 504) were selected with a stability score threshold being 0.8 (Figure 3A). The features were composed of 5 parts: (1) dose; (2) VHS; (3) demographic and anthropometrics features; (4) blood test features and (5) OCT features (Figure 3B). Based on the selected 25 features, a model was trained and optimized with a PCC of 0.649 in training set, which could predict test and validation set with a PCC of 0.638 and 0.685, respectively (Table 3). Thus, there was no obvious overfitting in our model.

**Table 3:** PCC of different models.

	**Training set (10×CV)**	**Test set**	**Validation set**
Original model	0.649	0.638	0.685
Model without blood test features	0.652	0.667	0.683
Model without blood test or OCT features	0.648	0.623	0.679

### Our Model Remained Good Performance Without Blood Test and OCT Features

**Figure 4:**
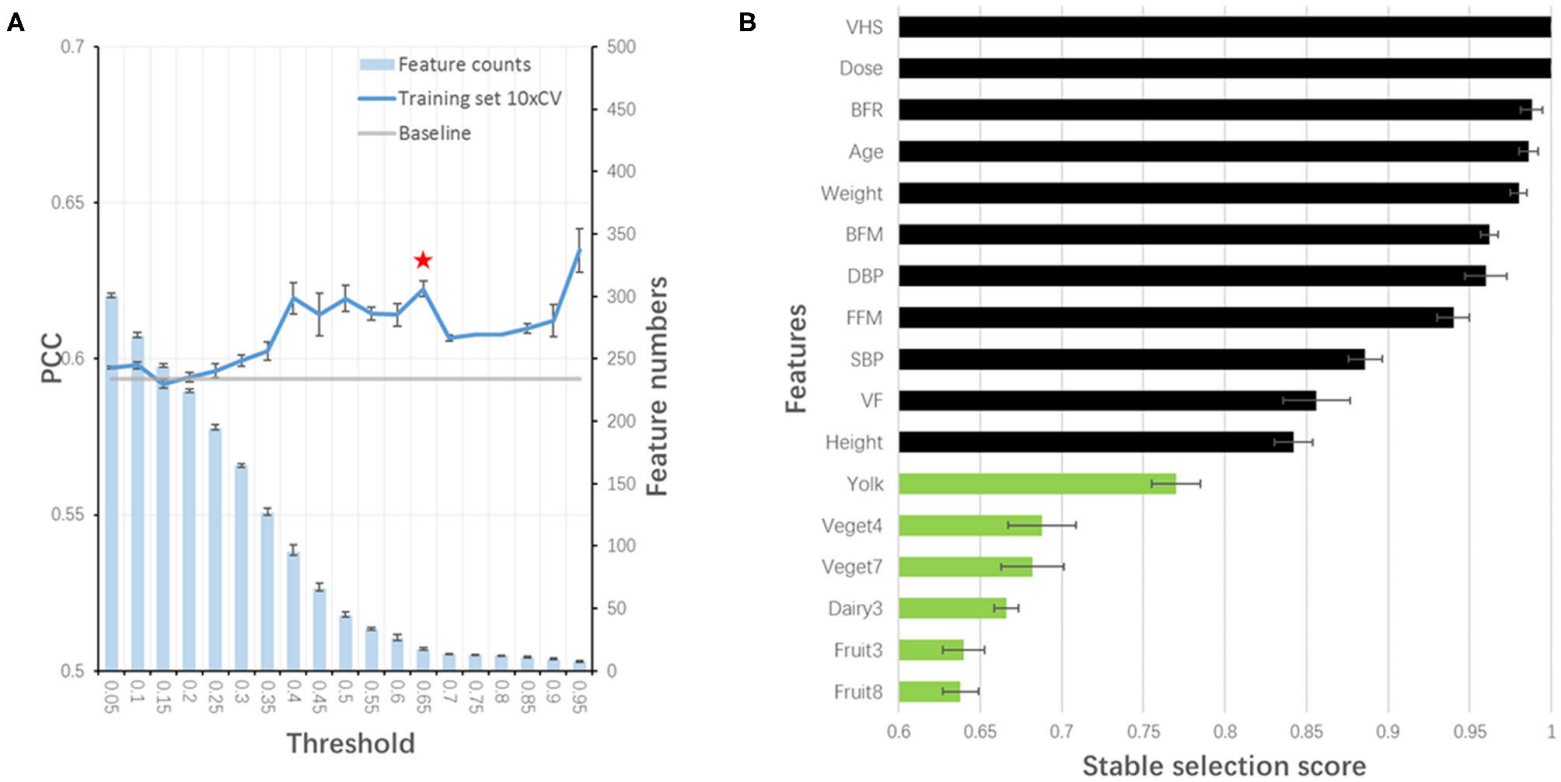
Stability-selection of features without blood test and OCT features. **(A)** Stability-selection with different threshold. Star denoted chosen threshold (0.65). Gray line: baseline PCC without stability-selection; blue line: 10xCV PCC in training set using different selected features; blue bar: feature numbers. **(B)** Selected features. Green, dietary features; black, other features.

Considering the feasibility of sample collection, we tried to train our model without blood test features. By redoing stability-selection, another 17 features (out of 447) were selected with the stability score threshold being 0.85. The model was trained and optimized, with a PCC of 0.652, 0.667, and 0.683 in training, test and validation set, respectively (Table 3). Considering the cost of sample collection, we additionally removed OCT features. Another set of 19 features (out of 413) was selected with the stability score threshold being 0.65 to train a new model, in which the PCC was 0.648, 0.623, and 0.679 in training, test and validation set, respectively (Table 3, Figure 4A). After the blood test and OCT features were excluded, both were replaced by dietary features (Figure 4B).

### Our Model Could Predict Personalized Dose for Each Subject

**Figure 5:**
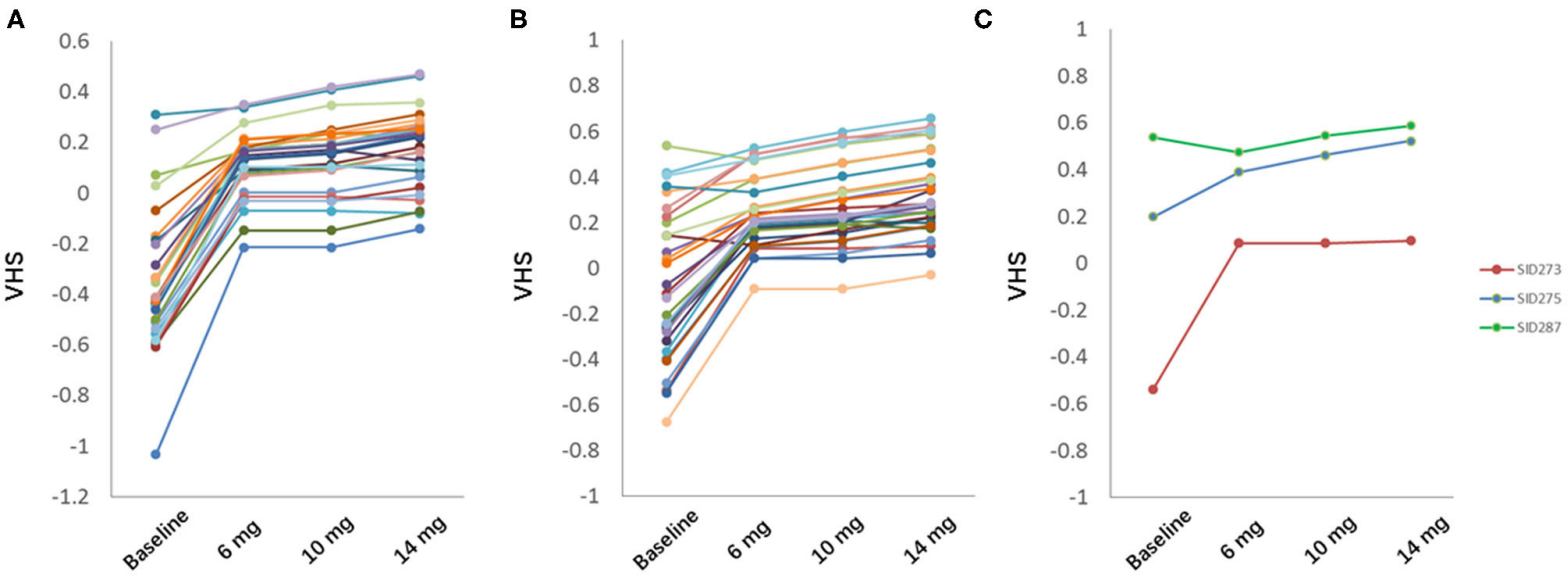
Prediction of optimal dose in test set **(A)** and validation set **(B)**. **(C)** 3 representative subjects in test and validation set. Green, eye fatigue showed no improvement; blue, eye fatigue showed significant improvement with highest dose; red, eye fatigue showed significant improvement with low or middle dose.

Among 28 subjects in test set and 30 in validation set, 56 subjects (96.6%) showed significant eye fatigue improvement with VHS elevated by more than 0.1 in 45 days, while other 2 subjects (3.4%) could not benefit from our botanical combination since they already had a relatively high VHS at baseline. 39 subjects (67.2%) should take 14 mg as the optimal dose, and other 17 subjects (29.3%) might take the combination at a lower dose level as the difference value of predicted VHS at V2 was <0.05 among 3 different doses (Figure 5).

## Discussion

Machine learning techniques have been well applied in medicine these years. Compared with traditional statistical models, they have many advantages including high power and accuracy, ability to model non-linear effects with more complex, high-dimensional and interactive variables, interpretation of large genomic data sets, robustness to parameter assumptions and dispense with normal distribution test (7, 13). Machine learning can be used for diagnoses of pediatric diseases, detection of upper gastrointestinal cancer combined with endoscopy, and prediction of mortality in patients with suspected coronary artery disease or rheumatoid arthritis (9, 10, 14, 15). For drug therapy, it is widely used to predict clinical response to antidepressant and optimal dose of warfarin (8, 16, 17). However, investigation on nutrition intervention is limited, especially lutein-related phytonutrients supplement.

Personalized nutrition received more and more attention these years. By utilizing machine learning algorithm of stochastic gradient boosting regression, glycemic responses to different types of food was predicted by Zeevi's group (18). In our study, hundreds of features, including anthropometrics, dietary habits, blood biomarkers, and eye-related indexes, were collected, and eye fatigue improvement was successfully predicted using XGBoost algorithm with PCC of training set 10xCV=0.618, which showed better performance than AdaBoost and ElasticNet algorithms. Upon feature selection and parameter optimization, the PCC was elevated to 0.649, which was comparable to that (0.68) in Zeevi's study. More importantly, using this validated model, we could predict the optimal dose of the botanical combination of lutein supplements for subjects with eye fatigue symptom and provide them with personalized nutrition solution. In our cohort, a certain number of subjects (67.2%) could benefit from the highest dose, some (29.3%) need lower doses to receive efficient supplement, and a small proportion (3.4%) could not benefit from the intervention. It suggested the rationality and necessity of personalized nutrition solution for phytonutrient supplementation.

To predict the eye fatigue improvement, VHS was developed from 4 eye-related features, including TSEFS, VP, MPOD, and ST to represent an overall eye fatigue level. Therefore, the top 2 features in our selected feature list were VHS and dose regardless of blood test and OCT features. Age was reported to be significantly correlated with eye fatigue in both children and white-collar worker with visual display units (19, 20). In particular, age-related macular degeneration could be prevented by lutein supplement, potentially attributed to the elevation in MPOD (21, 22). Age was another selected feature in our study. It was reported that there was a significant inverse relationship between the percentage of body fat and MPOD (23, 24). Body fat loss was positively related with the increased serum concentrations of lutein and zeaxanthin (25). In addition, HDL was significantly related to MPOD, serum lutein and zeaxanthin (26). So anthropometric features, such as BFR, BFM, FFM, and weight, and blood biomarkers, such as HDL, LDL, and TG, were selected for model development. Lutein and zeaxanthin are found in relatively high concentrations in leafy-green vegetables and brightly colored fruits. Cruciferous vegetables, such as kale and broccoli, and spinach are good dietary source (27). In addition, consumption of one egg per day increased serum lutein and zeaxanthin concentrations due to high bioavailability of lutein in the yolk (28). After blood test and OCT features were excluded, both were replaced by dietary features in the selected feature list.

Drawing blood is an invasive method of sample collection, and always leads to incompliance of the individuals. OCT is a non-invasive, cross-sectional imaging technique to evaluate retinal structure but with high cost and limited application (29). Considering the feasibility and cost of sample collection, we excluded blood test and OCT features to train another model, and finally got comparable performance with the original one. This optimization would make the personalized nutrition solution much easier to be accepted by the consumers. Genotype should be considered as one of the most important features in the personalized nutrition (30). But to date, little is known about the relationship between eye fatigue and genotype. In addition, genotyping increases the cost and time, which is not as convenient, efficient and cheap as the collection of phenotypes, such as anthropometrics, questionnaires and point-of-care testing (POCT). Therefore, in this study, we did not collect genotype features. Even so, there was still a report showing that SNP variants in *BCMO1* and *CD36* were associated with plasma lutein concentration and MPOD in humans (31). It suggests potential application of genotype in personalized nutrition for eye health, which will be considered in our future studies.

Besides the deficiency of genotype features, there are still several limitations in our study. In this study, subjects were administered only 3 doses, which limited the predicted dose to three. And this is the flaw of the algorithm itself. The study is additionally limited by relatively small sample size, limited ophthalmic examination findings in the enrollment of the subjects, and lack of external validation for model development.

In spite of these limitations, the study has its superiority. First, we predicted exact dose instead of response to the intervention. Second, to our knowledge, this is the first study to utilize machine learning technologies to investigate nutrition intervention, particularly the phytonutrients supplementation, for eye health. Third, the feature collection is simple and easy. All features, including anthropometrics, questionnaires and POCT, can be collected in a friendly way by staff instead of experienced doctor. Last but not the least, with the established prediction model, we can provide an integrated personalized nutrition solution in combination with the launched botanical product to the consumers, shedding new light on the direct-selling business.

In conclusion, by selecting different types of features collected in the RCT and applying XGBoost algorithm, we developed a model for predicting the optimized dose of the combination to provide personalized nutrition solution for consumer with eye fatigue in real world.

## Data Availability Statement

The datasets generated for this study are available on request to the corresponding author.

## Ethics Statement

The studies involving human participants were reviewed and approved by IRB of Shanghai Nutrition Society. The patients/participants provided their written informed consent to participate in this study.

## Author Contributions

JK and JD designed the research. AL and HZ conducted the research. JK, AL, and LC analyzed the data. JK and AL wrote the paper. JD had primary responsibility for final content. All authors read and approved the final manuscript.

## Conflict of Interest

The study design, implementation, analysis, and interpretation were carried out jointly by the funders. The study products were produced and supplied by Amway (China) R&D Center. JK, LC, and JD are employees of Nutrilite Health Institute, a division of Amway. The remaining authors declare that the research was conducted in the absence of any commercial or financial relationships that could be construed as a potential conflict of interest.
